# Machine learning-derived AS and AIS scores leverage BCAA metabolism and IL4I1 activity for prognosis and tailored therapy in ccRCC

**DOI:** 10.3389/fcell.2026.1720910

**Published:** 2026-02-25

**Authors:** Kang Qiang Weng, Xin Li, Xiao bao Chen, Jun wei Lin, Ling jun Liu, Le ye Yan, Ruo yun Xie

**Affiliations:** 1 Department of Urology, Fujian Medical University Union Hospital, Fuzhou, China; 2 Department of Interventional Radiology, Fujian Medical University Union Hospital, Fuzhou, China

**Keywords:** amino acid metabolism, clear cell renal cell carcinoma, IL4I1, machine learning, prognostic score

## Abstract

**Background and objective:**

Renal cell carcinoma (RCC) is among the most prevalent malignant tumors globally, characterized by a poor prognosis. The 5-year survival rate for advanced clear cell renal cell carcinoma (ccRCC) is below 20%.

**Materials and methods:**

This study utilized single-cell data analysis to examine the differences in branched-chain amino acid metabolism among ccRCC patients. Ten machine learning algorithms were employed to develop Amino acid Signature Score (AS score), integrating data from TCGA and GEO cohorts. We compared and validated the clinical characteristics, molecular features, and drug sensitivity of patients with varying AS scores. To address patient heterogeneity, principal component analysis was applied to construct an Amino acid Individualized Signature Score (AIS score) aimed at guiding personalized treatment and assessing its performance in immunotherapy and targeted therapy. Additionally, we explored the interaction between IL4I1 and branched-chain amino acid metabolism, along with the underlying causes of abnormal expression, using spatial transcriptomics and single-cell multi-omics approaches.

**Results:**

Branched-chain amino acid metabolism plays a crucial role in the progression and treatment of ccRCC. The AS score effectively distinguishes clinical characteristics and drug sensitivity across different patient subgroups. The AIS score confers a strategic advantage for second-line and immunotherapy when targeted therapy is ineffective. The elevated expression of IL4I1 enhances the degradation of branched-chain amino acids, promoting tumor growth and metastasis. Further analysis indicated that VHL mutations may elevate IL4I1 expression in tumors by modulating key transcription factors Hif-1a and SFMBT1, thus aggravating tumor progression.

**Conclusion:**

Branched-chain amino acid metabolism and IL4I1 are pivotal in the progression of ccRCC. AS classification and the AIS score present a robust framework for personalized treatment strategies, while IL4I1 shows potential as a novel therapeutic target to enhance treatment efficacy.

## 1 Introduction

Renal cell carcinoma (RCC) is the most aggressive malignant tumor of the urinary system, with a global incidence and mortality that continue to rise, making it the leading cause of mortality among renal tumors (Capitanio et al., 2019; Barata and Rini, 2017; Hsieh et al., 2017). Among its subtypes, clear cell renal cell carcinoma (ccRCC) is the most prevalent, accounting for over 80% of renal malignant tumors. Together with papillary RCC (pRCC) and chromophobe RCC (chRCC), these three types constitute 90% of RCC cases (Moch et al., 2016). Despite advances in imaging techniques that have improved early detection and facilitated radical surgery, approximately 30%–40% of patients still progress to metastatic disease (Barata and Rini, 2017). Notably, 20%–30% of patients present with metastases at the time of diagnosis, leading to a stark decline in the 5-year survival rate to just 12% (Atkins and Tannir, 2018; Rodriguez-Vida et al., 2017).

ccRCC is typically characterized by the accumulation of lipids in the cytoplasm, predominantly composed of fatty acids (FAs), triglycerides, and cholesteryl esters (Linehan et al., 2019). While previous studies have largely focused on lipid metabolism dysregulation, increasing evidence indicates that reprogramming of amino acid metabolism also plays a critical role in the progression of ccRCC (Tan et al., 2023). Unlike lipid metabolism, amino acid metabolism exhibits significant heterogeneity across different cancers, with its uptake and utilization patterns influenced by oncogenic signals and the tissue of tumor origin (Mayers et al., 2016; Commisso et al., 2013). Branched-chain amino acids (BCAAs), as essential amino acids, are linked to various cancer phenotypes, and their metabolic abnormalities may promote disease progression by altering the tumor microenvironment (Zhang and Commisso, 2019; Finicle et al., 2018). Although the roles of BCAAs in certain malignant tumors, such as breast cancer, have been elucidated (e.g., BCAT1 drives tumor growth through the mTOR pathway), their regulatory mechanisms in ccRCC remain largely unclear (Zhang and Han, 2017; Hattori et al., 2017). Moreover, BCAA metabolism in cancer is complex and context-dependent. In other malignancies, circulating BCAA levels can either rise (e.g., in PDAC and breast cancer) (Zhang and Han, 2017; Katagiri et al., 2018) or fall (e.g., in HCC and NSCLC) (Watanabe et al., 1984; Mayers et al., 2016), reflecting diverse tumor metabolic demands. While BCAA supplementation may benefit patients during therapy, it might also fuel tumor growth if administered beforehand, presenting a clinical dilemma (Brown et al., 2026). This systemic complexity underscores the limitation of peripheral BCAA levels as a guide for therapy and highlights the critical need to decipher the intratumoral BCAA metabolic state. In ccRCC, a cancer defined by unique metabolic drivers like VHL loss and HIF activation, two fundamental questions remain: what is the comprehensive landscape of intratumoral BCAA metabolism and its clinical relevance, and how can we quantify it to stratify patients for personalized treatment? Addressing these questions is imperative to transcend the contradictions observed in other cancers and to develop precise, metabolism-based biomarkers for ccRCC.

**FIGURE 1:**
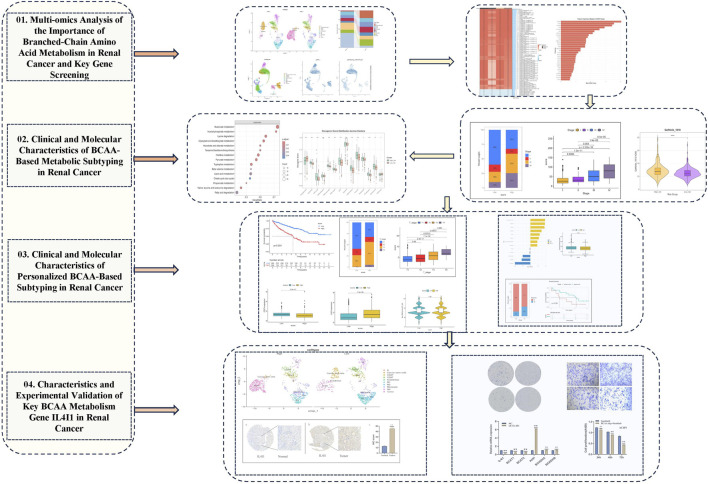
The work flow diagram of this study.

Recently, the advent of immunotherapy and targeted agents has significantly improved the median overall survival of patients with metastatic RCC. However, the heterogeneity of tumors leads to the differences in treatment response, which brings a major clinical challenge. Although there are methods to improve cancer treatment through the intratumoral microbiota and nanomedicins (Su et al., 2021; Zou et al., 2024), there is still an urgent need for new prognostic biomarkers to guide individualized treatment. In this study, we systematically reveal the critical role of BCAA metabolism in the progression of ccRCC and its response to therapy. Analysis of single-cell data has highlighted significant differences in BCAA metabolic profiles between patients with early-stage and late-stage disease, as well as immunotherapy responders. Based on these insights, we integrated 10 machine learning algorithms to develop an AS scoring system aimed at accurate classification of ccRCC patients. Furthermore, we constructed a personalized AIS score that demonstrated excellent performance in predicting responses to first-line, second-line, and immunotherapy. To elucidate the underlying mechanisms, we confirmed the oncogenic role of IL4I1 in ccRCC through functional experiments and analyzed its regulatory network using spatial transcriptomics and single-cell sequencing. These findings not only offer a new perspective on the metabolic heterogeneity of ccRCC but also lay a theoretical foundation for developing individualized treatment strategies based on BCAA metabolism. The research methodology is visually summarized in the Figure 1.

## 2 Materials and methods

### 2.1 Data source and preprocessing

The data for this study were obtained from a research article by Eliezer M. Van Allen (Bi et al., 2021), which analyzed single-cell data from three patients with clear cell renal cell carcinoma. The patients were divided into two groups: the first group consisted of untreated early (p90F_early_untreated) and late (p76F_late_untreated) stage patients; the second group included untreated patients with advanced disease ((p76F_late_untreated) and those with advanced disease who received combination immunotherapy with anti-PD-1 and anti-CTLA-4 (p915_late_Immunotherapy). The “scMetabolism” package was utilized to quantify the metabolic activities of the different groups, and the AUCell score was employed to analyze the differences in branched-chain amino acid (BCAAs) metabolism among various groups and cell subsets. To compare the expression of branched-chain amino acids across different stages and between the response and non-response groups of anti-PD-1 treatment, datasets GSE150404 and GSE67501 were downloaded from the GEO database.

### 2.2 Machine learning and SHAP analysis

To develop a prognostic model with high predictive accuracy, we followed these steps: (1) The TCGA-KIRC dataset was used as the training set. To ensure independent validation, we employed two separate external validation sets: GSE29609 and E-MTAB-1980. Furthermore, the TCGA-KIRC dataset was randomly split (50%/50%) to create an internal training subset and an internal validation subset for model development and preliminary evaluation. All feature selection and model training steps were confined exclusively to the TCGA internal training subset. A combination of machine learning algorithms was employed, including Lasso, Ridge, Elastic Net (Enet), Random Forest (RF), Stepwise Generalized Linear Model (Stepglm), Generalized Boosted Regression Model (GBM), Support Vector Machine (SVM), Extreme Gradient Boosting (XGBoost), glmBoost, and Naive Bayes. (2) All models were evaluated on the internal validation set, as well as on the two independent external validation sets (GSE29609 and E-MTAB-1980). (3) We calculated the area under the curve (AUC) and its 95% confidence intervals (CIs) for the TCGA and pooled cohorts, ranking all models according to the AUC index. (4) The top 31 potential signature genes were identified using the most efficient combination of algorithms. Notably, the Random Survival Forest (RSF) model demonstrated the best performance across the independent external validation sets (GSE29609 and E-MTAB-1980), and was therefore selected to construct the final prognostic signature, and interpretable SHAP (SHapley Additive exPlanations) analysis was conducted on the RSF model to enhance its interpretability by visually depicting how each feature influences the prediction results. Additionally, we utilized the GSCA database (https://guolab.wchscu.cn/GSCA/#/) to analyze branched-chain amino acid immune infiltration across different stages and examine changes in expression (Liu et al., 2023).

### 2.3 Constructing the amino acid signature (AS) score for stratification of renal cancer patients

We constructed the AS score using the Random Survival Forest (RSF) model for the stratified analysis of patients with ccRCC. The continuous AS score for each sample was defined as the predicted risk value generated by the RSF model, where a higher score indicates a poorer prognosis. To dichotomize patients into high- and low-risk groups, an optimal cutoff was determined using the maximally selected rank statistics method (via the surv_cutpoint function) solely within an internal validation set, and this fixed cutoff was then applied to all cohorts. Further analysis was performed to evaluate the high and low AS scores concerning clinical progression factors, and both univariate and multivariate regression analyses were conducted to assess the independent prognostic value of the AS score. Additionally, we investigated the relationship between the AS score and common first- and second-line clinical medications. Using the R packages clusterProfiler and GSVA, we analyzed the high and low molecular characteristics of the AS scores, and gene sets were downloaded from the CancerSEA database (http://biocc.hrbmu.edu.cn/CancerSEA) (Yuan et al., 2019).

### 2.4 Constructing the amino acid individualized signature (AIS) score to guide personalized treatment of patients with renal cell carcinoma

To provide a personalized treatment reference for renal cancer patients, we further constructed the AIS personalized score. First, differentially expressed genes between high and low AS scores were identified within the merged cohort. The expression matrix of these DEGs was subjected to principal component analysis (PCA), and the score of the first principal component (PC1) was extracted for each sample. To ensure a consistent directionality with clinical risk, a univariate Cox proportional hazards regression was performed on the PC1 scores. The final AIS score was calculated as: AIS Score = sign(β) × PC1, where β is the regression coefficient from the Cox model. Subsequently, the optimal cutoff to dichotomize patients into high- and low-risk AIS groups was determined using the maximally selected rank statistics method (via the surv_cutpoint function) and applied to the cohort.

Subsequently, associations between AIS scores and survival outcomes, as well as clinical factors and common immunotherapy targets, were assessed. The GSVA was utilized to evaluate the molecular signatures, metabolic profiles, and differences in cell death patterns between high and low AIS scores, with the corresponding gene sets downloaded from the MSigDB database. Simultaneously, the pRRophetic package was employed to compare the drug sensitivity of patients with high and low AIS scores concerning first-line and second-line drugs, including copper death inducers. Additionally, we collected data from two immunotherapy cohorts in the study by Riaz et al. (2017) to validate the predictive ability of the AIS score for immune response.

### 2.5 Spatial transcriptomics analysis of IL4I1 in clear cell renal cell carcinoma

Spatial transcriptomic data for ccRCC were obtained from the publicly available Gene Expression Omnibus (GEO) datasets GSE175540 and GSE179572. Specifically, we analyzed the following samples, identifiable by their GSM accession numbers: GSM5420752 (KIRC), GSM5924034 (KIRC6), GSM5924035 (KIRC7), GSM5924036 (KIRC8), GSM5924038 (KIRC10), and GSM5924049 (KIRC21). These datasets were generated using the 10x Genomics Visium platform with a spot diameter of 55 µm. For standardized data visualization and initial spatial analysis, we utilized the Sparkle web platform (https://www.grswsci.top), which integrates and re-analyzes published Visium data based on established methodologies (Shi et al., 2024; Xun et al., 2023). We emphasize that all underlying sequencing data are fully accessible and verifiable via the provided official GSM accession numbers on the NCBI GEO database.

The spatial analysis pipeline was conducted as follows. First, gene expression matrices for each tissue spot underwent quality control, normalization (analogous to Seurat::NormalizeData), and log-transformation. The predominant cell type for each spot (e.g., malignant cells, endothelial cells, macrophages) was then inferred via deconvolution analysis based on the expression of established cell-type signature genes within the platform (Shi et al., 2024; Xun et al., 2023). In this context, regions with high IL4I1 expression (“IL4I1-enriched regions”) were functionally defined by their spatial co-localization with and high expression within specific cell types of interest (namely, malignant cells and macrophages), rather than by applying an arbitrary expression threshold across all spots.

To visualize the spatial expression pattern of IL4I1, we employed the SpatialFeaturePlot function (from the Seurat package integrated into Sparkle), mapping its expression levels onto the physical coordinates of the tissue sections. For cross-sample comparison, the average expression of IL4I1 within spots annotated as the same cell type was calculated per sample. These average values were then Z-score normalized across samples to ensure comparability and visualized in a heatmap. To quantitatively assess spatial relationships, Spearman rank correlation analysis was performed between IL4I1 expression and the inferred abundance of specific cell types (e.g., CD8^+^ T cells, macrophages) across all spots within a tissue section. Correlation networks were visualized using the linkET R package. This entire analytical workflow was repeated across multiple independent samples (e.g., KIRC10 and KIRC21) to verify the consistency of the observed spatial expression patterns and associations.

### 2.6 Cell-cell interaction analysis of IL4I1 in clear cell renal cell carcinoma using single-cell RNA-seq data

Cell-cell communication analysis was performed on the matched single-cell RNA-seq data (GSE159115) using the CellChat R package (v1.6.0) and the CellChatDB.human ligand-receptor database (v1.0). Tumor cells were categorized as IL4I1-positive (IL4I1+) or IL4I1-negative (IL4I1-) based on detectable IL4I1 expression. The CellChat pipeline was applied to infer significant ligand-receptor-mediated interactions between cell groups. Communication probabilities were calculated, and their significance was assessed through 1,000 permutations, with p-values adjusted using the Benjamini-Hochberg method (FDR < 0.05). Networks for IL4I1+ and IL4I1- tumor cells were compared to identify interactions specific to the IL4I1+ subset, such as those involving the MIF and VEGF signaling pathways.

Simultaneously, we also downloaded the GSE167573, ICGC_EU, and GSE141577 datasets to explore the regulatory relationship of IL4I1. Additionally, we compared IL4I1 expression across multiple immunotherapy cohorts, including GSE135222, PMID: 20012197, and PMID: 30753825.

### 2.7 Correlation analysis and upstream mechanism of IL4I1 and BCAA related genes

In the previous analysis of single-cell data (Weng et al., 2024), we compared the effects of high and low IL4I1 expression on each cell subset in untreated patients and analyzed the molecular biological processes involved using the clusterProfiler package. Meanwhile, the metabolic pathways associated with high IL4I1 expression were evaluated using GSVA. Additionally, genes related to branched-chain amino acids in the IL4I1 transcriptome and proteome were analyzed using the PCA package, and the expression levels of these proteins in normal and tumor tissues were visualized. Simultaneously, the potential structural complexes of IL4I1 and its interacting proteins were analyzed with STRING.

To explore the mechanisms underlying the abnormal expression of IL4I1, pan-cancer e-QTL Bayesian co-localization analysis was performed using the coloc package to assess the probability that the two traits share the same causal variant. The cut-off value for co-localization evidence was set at a PP.H4.abf greater than 0.75. The gassocplot2 package was then utilized for visualization. Additionally, the mutation landscape of IL4I1 was visualized using the maftools package.

In the epigenetic analysis, TSS1500 (TSS upstream −200 to −1,500 bp), TSS200 (TSS upstream −200 bp), the first exon (1stExon), and the 5′UTR (5′untranslated region) were included in the methylation analysis. Bubble plots were used to visualize the differences in the methylation levels of a gene at different locations and in different cancer types. For transcription factor analysis, the position of the peak of gene ATAC-seq in the genome was visualized using the covplot function of the ChIPseeker package, and the correlation between transcription factor expression and the ATAC peak was quantified using the Spearman rank correlation coefficient. Furthermore, we utilized the GSE167573 dataset to verify the regulatory effect of SFMBT1 transcription factor knockdown on IL4I1 in ccRCC.

### 2.8 Cell, reagent, and tissue microarray

The human normal cell line HK-2 was purchased from Wuhan Procell Life Science and Technology Co., Ltd. Human cancer cell lines, including 786-O, ACHN, and 769-P, were obtained from the American Type Culture Collection (ATCC). All cell lines were cultured in DMEM/F-12 medium, supplemented with 10% fetal bovine serum (FBS) and 1% penicillin-streptomycin mixture. Prior to use, all cell lines were verified to ensure the absence of *mycoplasma* contamination and were cultured in a humidified incubator at 37 °C with 5% CO_2_.

We acquired a human clear cell renal cell carcinoma (ccRCC) tissue microarray (HKid-CRC060CS-01), which included tumor tissues and corresponding adjacent normal tissues, from Shanghai Xinchao Biotechnology Co., Ltd. Branched-chain amino acid (BCAA) deprivation experiments were conducted using customized BCAA-free conditioned medium (Living, cat: LVN1004-BCAA). Immunohistochemical staining was performed as previously described (Weng et al., 2024), utilizing immunohistochemical (IHC) analysis of patient tissue microarrays with the IL4I1 antibody (1:200, Abcam, ab176588).

### 2.9 CCK-8 experiment

The Cell Counting Kit-8 (#BS350BT) was purchased from Biosharp. Cells were cultured in normal medium and BCAA-free conditioned medium and were seeded into 96-well plates to assess cell proliferation ability at different time points. Additionally, the sensitizing effect of sunitinib in the presence of the drug was evaluated in BCAA-free conditioned medium, and the proliferation capacity of the cells was compared at different time points.

### 2.10 Construction of stable cell lines, plate cloning, and migration assays

IL4I1 knockdown. To silence IL4I1 expression, three distinct short hairpin RNA (shRNA) sequences targeting different regions of the IL4I1 transcript (NM_152899.2) were used (Target a, b, c; sequences listed in Supplementary Figure S3). Each shRNA was individually cloned into a pLenti-U6-shRNA-CMV-GFP-2A-Puro lentiviral vector. For the experimental group, lentiviruses carrying these three shRNAs were pooled at equal titers to transduce cells, establishing a stable polyclonal IL4I1-knockdown population (shIL4I1). A lentivirus expressing a non-targeting scrambled shRNA (sequence in Supplementary Figure S3) was used to generate the control cell line. The efficiency of IL4I1 knockdown was confirmed by quantitative RT-PCR (RT-qPCR) analysis.

Lentiviral transfection was performed according to the manufacturer’s instructions (Applied Biological Materials Inc., Vancouver, BC, Canada). Following transduction, stable expression cell lines were established via selection with puromycin. For the colony formation assay, the respective cell lines were seeded in triplicate into six-well plates and cultured for 14–21 days to allow colony formation. The resulting colonies were then stained and quantified using crystal violet (Beyotime, cat: C0121-500 mL). For the cell migration assay, cells were seeded in triplicate into the upper chamber of a 24-well plate, while the lower chamber was filled with 600 μL of medium containing 10% serum. After a 12-h incubation at 37 °C, the cells were fixed with 4% paraformaldehyde for 30 min, followed by crystal violet staining and quantitative analysis.

### 2.11 Regulation of branched-chain amino acid catabolism by knockdown of IL4I1

Total RNA was extracted from cells using the Trizol method (Invitrogen, Carlsbad, CA, United State), and cDNA synthesis and qPCR amplification were performed using HiScript III RT SuperMix. PCR amplification was conducted using ChamQ Universal SYBR qPCR Master Mix (Vazyme, Nanjing, China) according to the manufacturer’s instructions. The primers used in this study are detailed in the Supplementary File.

### 2.12 ELISA analysis of BCKA content at different time points

Branched-chain keto acid (BCKA) content was measured at different time points following the knockdown of IL4I1 using an ELISA kit according to the manufacturer’s instructions. The human BCKA ELISA kit was purchased from Mlbio (catalog number: #YJ912536).

### 2.13 Statistical analysis

Data analyses and presentations were conducted using R software (version 4.3.1) and Python (version 3.10.11). Results are expressed as mean ± standard deviation. Student’s t-test or Mann-Whitney U test was used for two-group comparisons, while one-way ANOVA or Kruskal-Wallis H test was used for multiple group comparisons. Kaplan-Meier survival differences were assessed with the log-rank test. Statistical significance was defined as P < 0.05. Part of the flow chart was drawn on the online Platform GDP (BioGDP - Generic Diagramming Platform for Biomedical Graphics) (Jiang et al., 2025).

## 3 Result

### 3.1 Importance of branched-chain amino acid metabolism in clear cell renal cell carcinoma

**FIGURE 2:**
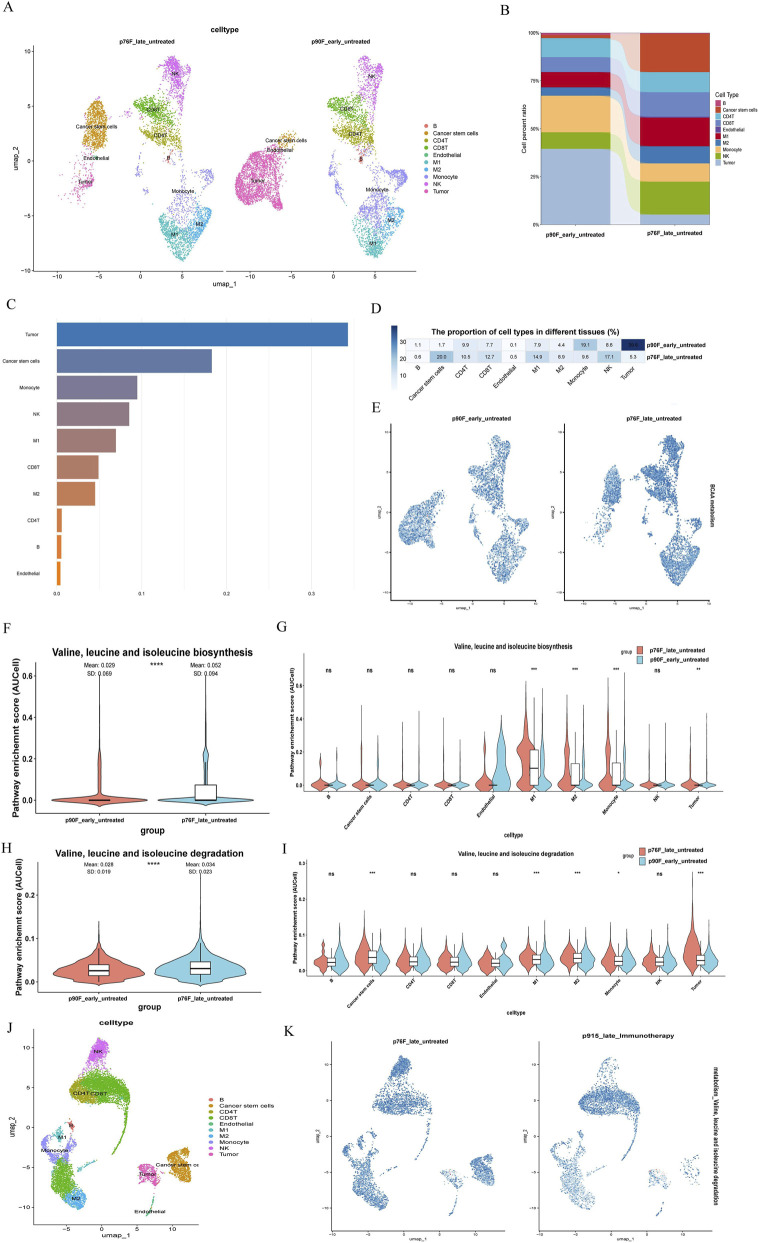
Importance of branched-chain amino acid metabolism in clear cell renal cell carcinoma. **(A)** Cell type marker genes displaying single-cell data. Visualization of UMAPs for different cell types in the early (right) and late (left) groups. **(B)** Proportions of different cell types in the early and late stages, represented by bars. **(C)** Bar graph illustrating cell types exhibiting the largest changes. **(D)** Heatmap displaying the differences among each type of cell subset across different groups. **(E)** Elevated BCAA metabolism in late-stage untreated ccRCC (UMAP). **(F)** Between-group differences in branched-chain amino acid (BCAA) synthesis during early and late stages. **(G)** Metabolic differences among each subgroup regarding BCAA synthesis in early and late stages. **(H)** Group differences in early and late BCAA degradation. **(I)** Metabolic differences among each subgroup concerning BCAA degradation in early and late stages. **(J)** UMAP representation of advanced untreated renal cancer. **(K)** Differences in BCAA degradation metabolism between treated (right) and untreated (left) late-stage groups.

We compared single-cell data from the early and advanced stages of annotated ccRCC (Figure 2A), examining expression differences among various cell subsets (Figure 2B) and analyzing the magnitude and proportion of changes in each subset (Figures 2C,D). To directly assess the functional metabolic alterations, we calculated the BCAA metabolic activity score using AUCell analysis. This revealed a significant increase in BCAA metabolism in late-stage compared to early-stage tumors (Figure 2E). Consequently, we focused on assessing the differential expression of branched-chain amino acid (BCAA) synthesis across groups (Figure 2F) and its impact on individual cell subsets (Figure 2G). Additionally, we analyzed the between-group differences in BCAA degradation between stages (Figure 2H), alongside its expression variation within each cell subset (Figure 2I). Our findings reveal significant differences in BCAA metabolism within the tumor tissue of patients at different stages, suggesting a marked increase in BCAA metabolism specifically in myeloid cells and tumor cells of late-stage patients. We also compared BCAA metabolism in late-stage patients receiving immunotherapy (without concurrent targeted therapy), and observed that within the treatment group, BCAA metabolic activity decreased in NK cells, myeloid cells, and tumor cells, while it increased in T cells (Figures 2J,K). These results indicate that branched-chain amino acid metabolism may play an important role in the advanced stage and treatment context of ccRCC.

### 3.2 Prognostic model construction of BCAA metabolism in ccRCC based on machine learning

**FIGURE 3:**
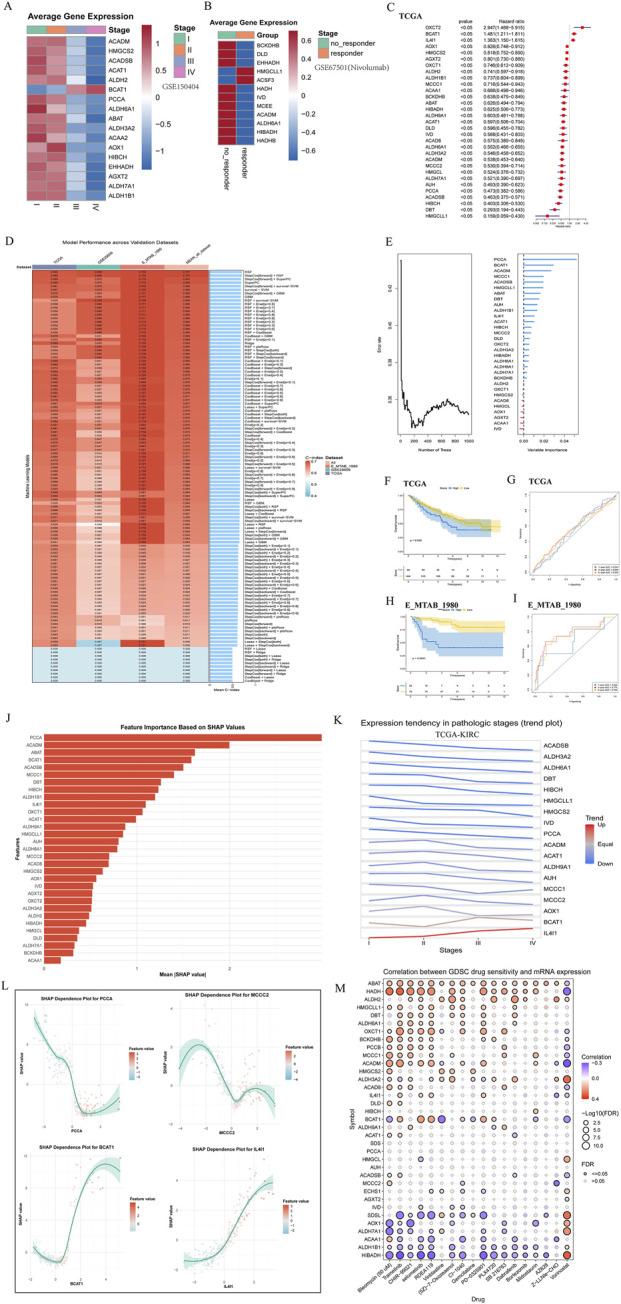
Prognostic model construction of BCAA metabolism in ccRCC based on machine learning. **(A)** mRNA expression differences of branched-chain amino acid metabolism-related genes across different stages in GSE150404. **(B)** Differential mRNA expression of branched-chain amino acid metabolism genes in patients from GSE67501 who responded to anti-PD-1 therapy compared to non-responders. **(C)** Results of univariate prognostic regression analysis of differentially expressed genes related to branched-chain amino acid metabolism, combining data from TCGA-KIRC. **(D)** Ten machine learning algorithms and various combinations were employed to construct prognostic models, with resulting concordance indices presented. **(E)** The Random Survival Forest (RSF) model was used to analyze the importance of 31 genes. **(F)** Survival analysis of theTCGA-KIRC. **(G)** Time-dependent ROC curves for 1-, 3-, and 5-year overall survival prediction in the TCGA-KIRC. **(H)** Survival analysis of E-MTAB-1980. **(I)** Time-dependent ROC curves for 1-, 3-, and 5-year overall survival prediction in the E-MTAB-1980 validation set. **(J)** SHAP analysis of the RSF model, depicting the distribution of SHAP values among key feature genes, emphasizing the varying impacts of individual genes on model predictions. **(K)** Expression trends of model genes across different stages in TCGA-KIRC. **(L)** The SHAP dependence diagram illustrates the relationship between feature values and SHAP values. **(M)** Correlation between branched-chain amino acid metabolism genes and immune cell infiltration in clear cell renal cell carcinoma.

We further analyzed the expression differences of branched-chain amino acid (BCAA) metabolic-related genes across various stages in the transcriptome and found that most of these genes were highly expressed in the early stage and exhibited lower expression in the late stage, with the exception of BCAT1 (Figure 3A). Additionally, we compared the expression of metabolism-related genes between responders and non-responders in the PD-1 immunotherapy cohort. Notably, most responders showed low expression of metabolic genes, with ACSF3 and HMGCLL1 being exceptions that exhibited high expression (Figure 3B).We screened genes associated with BCAA metabolism to identify prognostic factors in the combined cohort, discovering that most were risk factors; however, OXCT2, BCAT1, and IL4I1 were identified as protective factors (Figure 3C).

We employed 108 combinations of 10 machine learning algorithms for variable selection and model development of Amino acid Signature (AS). The TCGA-KIRC cohort was partitioned into a training set and an internal validation set. The trained models were then rigorously assessed on this held-out internal validation set, as well as on two entirely independent external cohorts (GSE29609 and E-MTAB-1980). Among all algorithms evaluated, the Random Survival Forest (RSF) model consistently demonstrated superior and robust prognostic performance across both the internal and external validation sets (Figure 3D). The RSF model demonstrated highly stable predictive 317 performance across all validation sets, with C-index values of 0.689 (TCGA-valid), 0.696 318 (GSE29609), and 0.735 (E-MTAB-1980)—yielding a coefficient of variation of only 3.0%. 319 Moreover, it consistently ranked first in both external cohorts. This reproducibility, along with the 320 minimal difference in average C-index between RSF and the next-best model, confirms that the 321 selection reflects robust, top-tier performance rather than a chance advantage in any single dataset. We subsequently established an RSF model incorporating these 31 genes and illustrated their relative importance (Figure 3E), with PCCA, AUH, and others showing significant influence.

It is crucial to clarify that the AS score is not a direct linear measure of BCAA metabolic pathway activity. Rather, it functions as an integrative prognostic signature. A high AS score operationally defines a tumor molecular subtype characterized by “BCAA metabolic reprogramming,” which is collectively associated with an unfavorable prognosis. Consistent with this, survival analysis of the TCGA-KIRC (Figure 3F) demonstrated that patients with higher AS scores experienced worse prognoses. Time-dependent AUC values indicated robust predictive accuracy at 1, 3, and 5 years (Figure 3G). Higher AS scores were consistently associated with poorer survival outcomes across cohorts (Figures 3F,H,I).Furthermore, we conducted SHAP analysis on the RSF model, revealing the distribution of SHAP values for key feature genes (Figure 3J). This analysis highlighted the contributions of different genes to model predictions, with the top five influential genes being ACADM, PCCA, ACADSB, IL4I1, and ALDH3A2. We explored the expression trends of these genes across various stages in TCGA-KIRC (Figure 3K), noting that IL4I1 expression progressively increased with advancing stage.

Moreover, we examined the relationship between expression levels and SHAP values. A higher SHAP value correlated with a greater likelihood of poor prognosis, with IL4I1 showing a significant effect; its expression level was positively associated with the probability of poor outcomes (Figure 3L). Importantly, high expression of IL4I1 was positively correlated with sensitivity to Trametinib and Selumetinib, but negatively correlated with sensitivity to Vorinostat and Z-LLNle-CHO (Figure 3M).

### 3.3 Clinical features associated with different AS score

**FIGURE 4:**
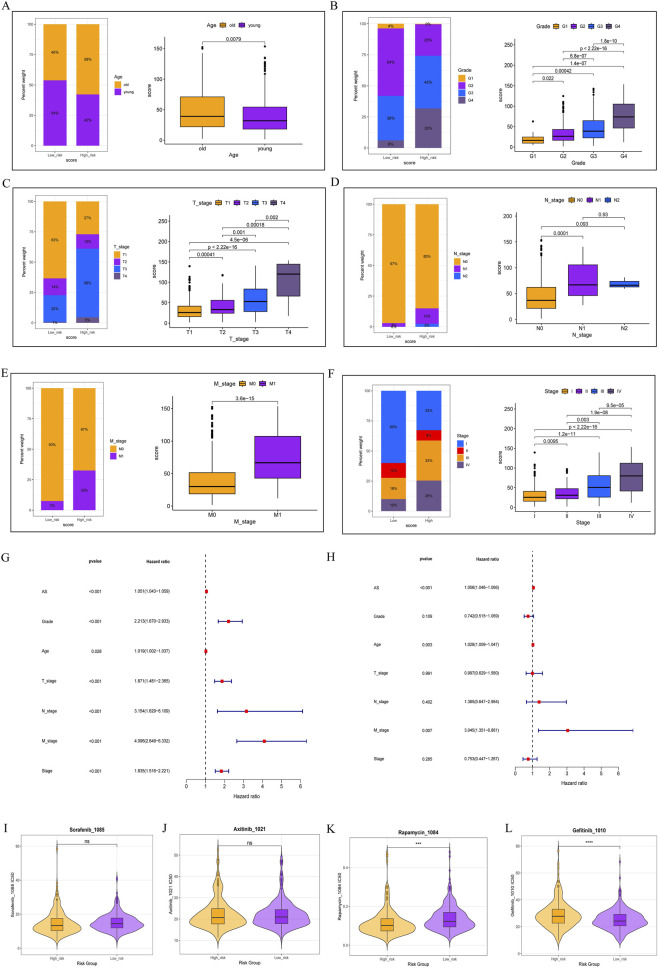
Patients were stratified into high and low groups based on Amino acid Signature Score (AS score). Proportions of different scores with clinical relevance are depicted on the left, while expression differences are shown on the right. **(A)** Relationship between high and low scores and patient age. **(B)** Association between high and low scores and pathological grade. **(C)** Relationship between high and low scores and clinical stage T. **(D)** Relationship between high and low scores and clinical stage N. **(E)** Relationship between high and low scores and clinical stage M. **(F)** Relationship between high and low scores and overall clinical stage. **(G)** Univariate regression analysis of high and low scores in clear cell renal cell carcinoma. **(H)** Multivariate regression analysis of high and low scores in clear cell renal cell carcinoma, indicating negative results. **(I)** Sensitivity differences to sorafenib between high and low score groups. **(J)** Sensitivity differences to axitinib between high and low score groups. **(K)** Sensitivity differences to rapamycin between high and low score groups. **(L)** Sensitivity differences to gefitinib between high and low score groups.

Based on the AS score, ccRCC cohort was divided into two groups, allowing for a more detailed exploration of the clinical characteristics within the AS subgroups. Patients in the high-score group constituted a significant proportion of the elderly population (Figure 4A). Among the clinical progression factors, the high-score group exhibited a strong association with pathological grade, T, N, M classification, and overall stage, indicating a positive correlation with these progression factors (Figures 4B–F). Furthermore, both univariate (Figure 4G) and multivariate regression analyses (Figure 4H) suggested that the AS score serves as an independent risk factor. We also evaluated the predicted drug sensitivity based on the AS score to first-line and second-line drugs in ccRCC. In the high-score group, patients showed increased predicted sensitivity to sorafenib, axitinib, and rapamycin (Figures 4I–K). Conversely, gefitinib demonstrated reduced predicted sensitivity in the high-score group (Figure 4L).

### 3.4 Differences in tumor biology between patient subgroups with high and low scores

**FIGURE 5:**
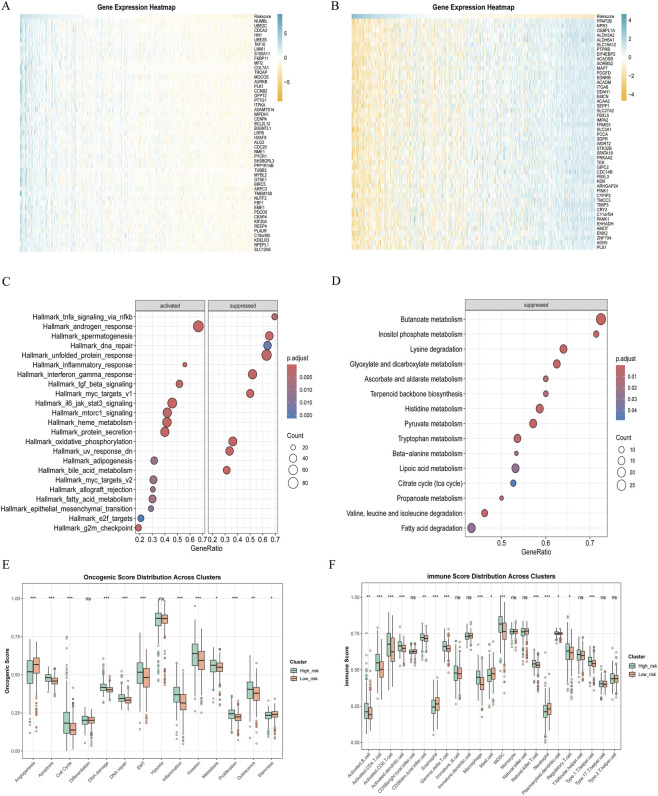
The subgroups (high vs. low) were defined by on Amino acid Signature Score (AS score). **(A)** Correlation analysis of amino acid model scores with all genes, presented as a heatmap showing the top 50 positively correlated genes. **(B)** Expression levels of the top 50 negatively correlated genes. **(C)** GSEA was performed to assess differences in enrichment pathways between the high-score and low-score groups. **(D)** Comparison of metabolic enrichment differences between the high-score and low-score groups. **(E)** Phenotypic relationships between patient subgroups with high and low scores and tumor progression. **(F)** Relationship between patient subgroups with high and low scores and tumor immune infiltration.

We further analyzed the molecular biological differences among AS subtypes. Genes positively and negatively correlated with the AS score were evaluated (Figures 5A,B). Gene Set Enrichment Analysis (GSEA) revealed that the high-score group activated pathways associated with the JAK-STAT3 chronic inflammatory response, fatty acid metabolism, and epithelial-mesenchymal transition, while inhibiting the NF-κB acute inflammatory response pathway and the unfolded protein response (Figure 5C). Patients in the high-score group exhibited inhibition of various metabolic activities, including branched-chain amino acid degradation, fatty acid degradation, and the tricarboxylic acid cycle (Figure 5D).

Regarding tumor progression phenotypes, we observed that high score patients exhibited inhibition of angiogenesis and tumor stemness, while promoting apoptosis, cell cycle progression, DNA damage response, inflammation, and epithelial-mesenchymal transition (Figure 5E). In terms of immune infiltration, patients with high scores were primarily associated with B cells, CD4^+^ T cells, and CD8^+^ T cells. Among myeloid cells, macrophages were the predominant infiltrating cell type (Figure 5F).

### 3.5 Construction of personalized scores for subgroups

**FIGURE 6:**
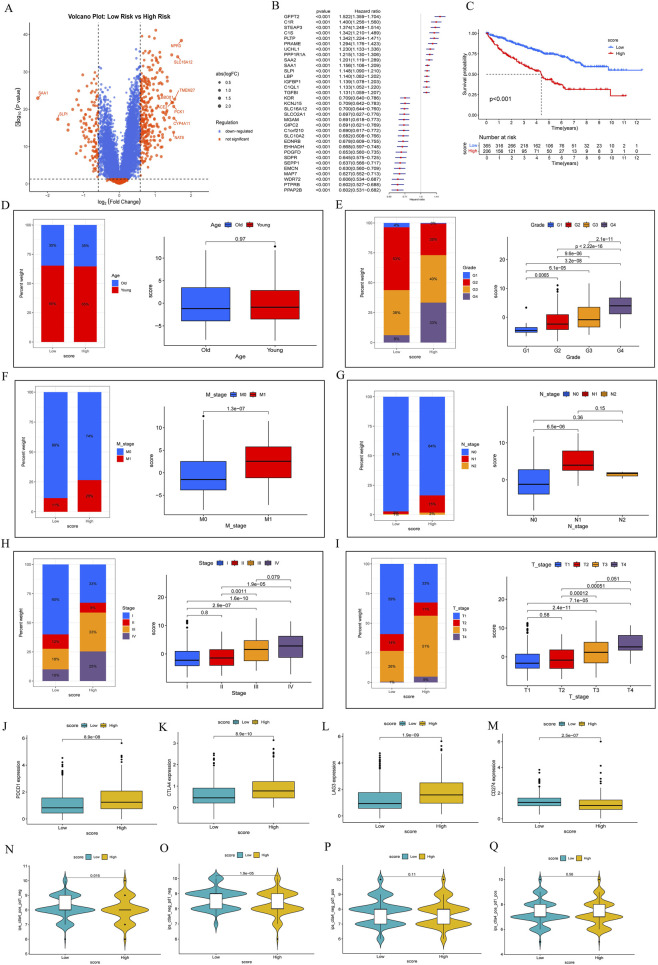
Patients were stratified into high and low groups based on Amino acid Individualized Signature Score (AIS score) constructed in this analysis. **(A)** Volcano plot showing differentially expressed genes between high and low score subgroups. **(B)** Prognostic regression analysis of differentially expressed genes in subgroups. **(C)** Prognostic curves for personalized scores. **(D)** Relationship between personalized scores and age. **(E)** Relationship between personalized scores and pathological grade. **(F)** Relationship between personalized scores and clinical stage M. **(G)** Relationship between personalized scores and clinical stage N. **(H)** Relationship between personalized scores and clinical stage (Stage). **(I)** Relationship between personalized scores and clinical stage T. **(J)** Expression differences of PDCD1 at different scores. **(K)** Expression differences of CTLA4 at different scores. **(L)** Expression differences of LAG3 at different scores. **(M)** Expression differences of CD274 at different scores. **(N)** Differences in IPS scores among CTLA4-positive, PD1-negative patients with different scores. **(O)** Differences in IPS scores among CTLA4-negative, PD1-negative patients with different scores. **(P)** Differences in IPS scores among CTLA4-negative, PD1-positive patients with different scores. **(Q)** Differences in IPS scores among CTLA4-positive, PD1-positive patients with different scores.

To provide more precise, personalized therapeutic guidance, we sought to achieve finer stratification within the patient cohort. Specifically, within the high-risk subgroup defined by the AS score, we aimed to construct a second-level Amino acid Individualized Signature (AIS). The AIS score is designed to capture the residual heterogeneity among high-AS patients, with a higher AIS score identifying a subset that possesses more extreme biological features and differential therapeutic vulnerabilities. Accordingly, we analyzed the differentially expressed genes between the two patient subgroups (Figure 6A). Prognostic regression analysis of these differentially expressed genes revealed that C1QL1, C1R, C1S, SAA1, and WDR72 were identified in previous studies (Figure 6B) (Weng et al., 2024). Specifically, C1QL1, C1R, C1S, and SAA1 were found to be protective factors, while WDR72 was identified as a risk factor. The personalized score indicated that higher scores were associated with poorer prognosis (Figure 6C).

We established an AIS score for the differentially expressed genes using principal component analysis. In terms of clinical correlation, there was no significant difference in AIS scores when stratified by age (Figure 6D). Regarding clinical progression, the high-score group was closely linked to pathological grade, T, N, M classifications, and overall clinical stage, exhibiting positive correlations with these progression factors (Figures 6E–I).

Furthermore, we analyzed the expression differences of common immune checkpoints between high and low AIS score groups. The expression levels of PDCD1, CTLA4, and LAG3 were relatively higher in the high-score group (Figures 6J–L), while the expression of CD274 was relatively lower in this group (Figure 6M), suggesting a potential difference in the immune microenvironment. Additionally, we examined the Immunophenoscore (IPS) response scores between PD1-negative and PD1-positive groups. Among PD1-negative patients, those with positive CTLA4 scores exhibited lower IPS values (Figure 6N), whereas those with negative CTLA4 scores had higher IPS values (Figure 6O). No significant differences were observed between PD1-positive patients in relation to CTLA4 scores and IPS (Figures 6P,Q).

### 3.6 Oncology differences in AIS scores

**FIGURE 7:**
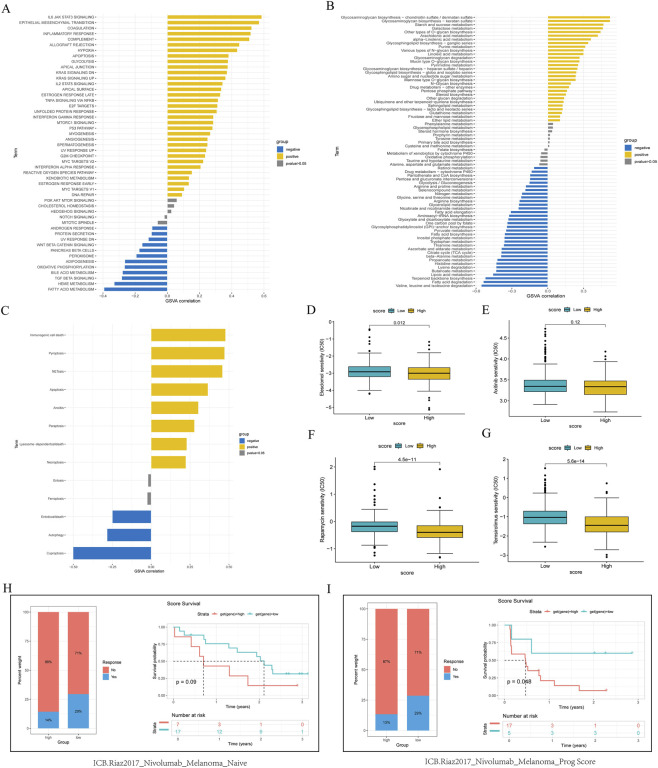
The subgroups were defined by amino acid individualized signature score. **(A)** Transcriptional pathway analysis for high AIS scores. **(B)** Metabolic enrichment analysis for high AIS scores. **(C)** Analysis of various cell death patterns associated with high AIS scores. **(D)** Sensitivity analysis of Elesclomol across different AIS scores. **(E)** Sensitivity of axitinib in relation to different AIS scores. **(F)** Sensitivity of rapamycin with respect to different AIS scores. **(G)** Sensitivity of Temsirolimus to varying AIS scores. **(H)** Treatment response (left) and prognosis curves (right) for different AIS scores in the cohort not treated with nivolumab. **(I)** Treatment response (left) and prognosis curve (right) based on AIS score in the cohort receiving nivolumab.

We further analyzed the individual oncological differences based on AIS scores and found that the high AIS score group activated chronic inflammation, promoted epithelial-mesenchymal transition, and enhanced glycolysis. In contrast, it inhibited fatty acid metabolism and the Wnt/β-catenin pathway (Figure 7A). The high-score group also promoted the synthesis of N-glycosylation and O-glycosylation while inhibiting the degradation of fatty acids and branched-chain amino acids (Figure 7B). Additionally, we evaluated the differences in AIS scores through cell death mode analysis and found that, intriguingly, the high score group inhibited copper-induced cell death (Figure 7C). We further compared the expression of Elesclomol, a copper death inducer, across the scores, finding that higher AIS scores were associated with increased predicted drug sensitivity (Figure 7D). Notably, there was no significant difference in the predicted response to axitinib, a common targeted agent, between the two groups (Figure 7E). For second-line agents, mTOR inhibitors rapamycin and Temsironlimus showed higher sensitivity (Figures 7F,G).

In evaluating immune efficacy, we analyzed treatment responses of different AIS scores in a cohort without nivolumab treatment. The high AIS score group exhibited a poor immune response, and a notable difference in prognosis was observed between the two groups (Figure 7H). In the cohort receiving nivolumab treatment, the high AIS score group demonstrated significantly poorer immune efficacy (Figure 7I). These exploratory findings suggest a potential link in melanoma; however, as the analysis was not performed in a ccRCC-specific immunotherapy cohort, the results require validation and should not be directly extrapolated to predict clinical benefit in ccRCC.

### 3.7 Key marker genes of IL4I1 in ccRCC

**FIGURE 8:**
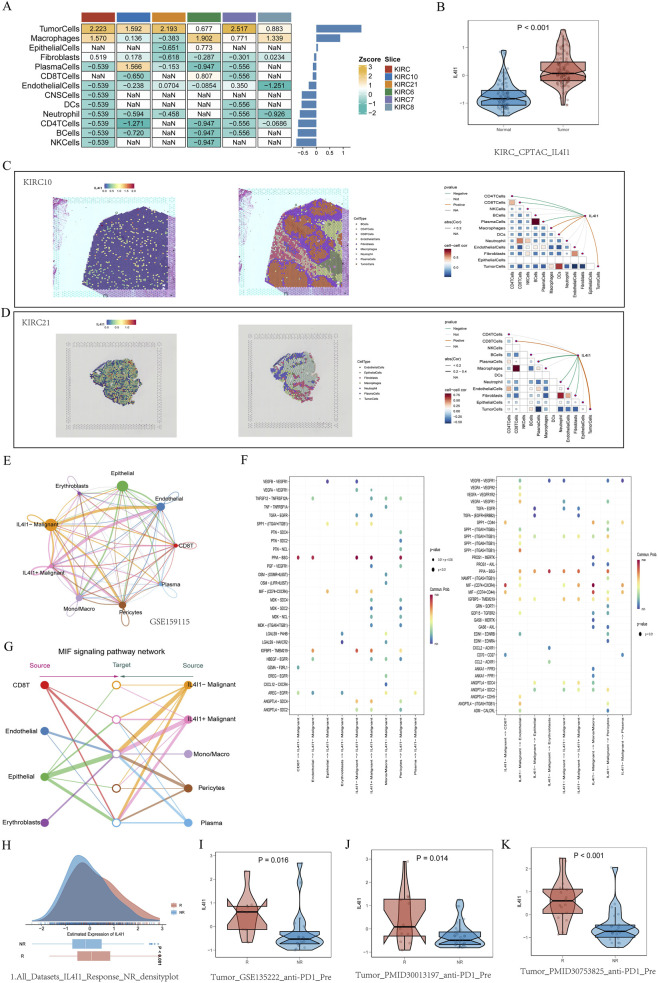
Key marker genes of IL4I1 in ccRCC. **(A)** Heatmap depicting IL4I1 expression across different cell types in spatial transcriptome sections of KIRC. **(B)** Proteomic differences in IL4I1 levels between normal and tumor samples from the CPTAC cohort. **(C)** Correlation analysis of IL4I1 with various cell types in KIRC10. (Left) Showcasing the localization of IL4I1 expression within individual subsets. (Middle) Identification of each subgroup. (Right) Correlation between IL4I1 and specific immune cells. **(D)** Correlation analysis of IL4I1 with different cell types in KIRC21. (Left) Expression localization of IL4I1 within each subset. (Middle) Identification of each subgroup. (Right) Correlation between IL4I1 and distinct immune cells. **(E)** Venn diagram illustrating interactions between the IL4I1 tumor subpopulation and other cellular subpopulations in GSE159115. **(F)** Interaction dynamics of various cell subsets with IL4I1 tumor subsets. **(G)** Role of MIF signaling in the interactions among cell subsets. **(H)** Differential expression of IL4I1 in the immunotherapy cohort. **(I)** Differential IL4I1 expression in the PD-1 immunotherapy cohort at GSE135222. **(J)** Differential expression of IL4I1 in the immunotherapy cohort described in PMID30013197. **(K)** Differential expression of IL4I1 observed in the immunotherapy cohort studied in PMID30753825.

In our analysis of spatial transcriptome sections of ccRCC, we focused on the expression pattern of the IL4I1 gene, as depicted in the expression heatmap across different cell types in KIRC spatial transcriptome sections (Figure 8A). IL4I1 was predominantly expressed in tumor cells and macrophages, showing low expression levels in most immune cells. Additionally, CPTAC-KIRC proteomics data corroborated that IL4I1 is highly expressed in tumor tissues (Figure 8B). We further analyzed the correlation between IL4I1 expression and the infiltration of various immune cells in the corresponding spatial transcriptome sections. IL4I1 exhibited a positive correlation with immune infiltration by tumor cells and macrophages, while showing a negative correlation with factors such as CD8T cells (Figure 8C). A similar pattern was observed in another patient, demonstrating a strong correlation with tumor cells and a positive correlation with CD8T cell infiltration (Figure 8D).

We also examined the cellular interactions between IL4I1-negative tumor subsets and other cellular populations, particularly those related to myeloid cells and endothelial cells (Figure 8E). MIF (CD74+CXCR4) plays a critical role in facilitating communication between IL4I1 tumor cells and both CD8T cells and macrophages, while VEGF-VEGFR1 is pivotal for interactions between IL4I1 tumor cells and endothelial cells. This suggests a potential utility for IL4I1 in targeted therapy and immunotherapy (Figure 8F). Furthermore, we demonstrated the involvement of MIF signaling in these cellular interactions (Figure 8G). Additionally, we analyzed the differential expression of IL4I1 in the immunotherapy cohort, revealing high expression levels in groups that responded to immunotherapy (Figure 8H). Our validation in additional immunotherapy cohorts confirmed elevated IL4I1 expression across multiple groups (Figures 8I–K). Hence, IL4I1 emerges as a potential biomarker for predicting immunotherapy response.

### 3.8 Knockdown of IL4I1 inhibits tumor proliferation and growth by disrupting BCAA catabolic homeostasis

**FIGURE 9:**
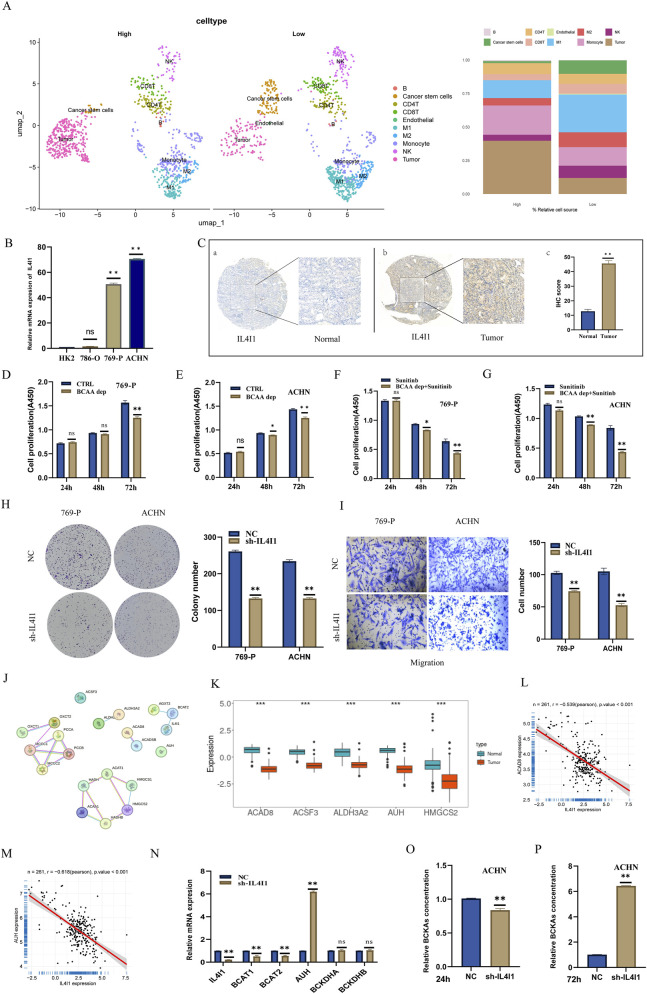
Knockdown of IL4I1 inhibits tumor proliferation and growth by blocking the catabolism of branched-chain amino acids (BCAA). **(A)** Impact of high and low IL4I1 expression on each cell subpopulation, presented as a UMAP plot on the left, with bar graphs on the right showing the proportional changes in each subgroup. **(B)** Expression levels of IL4I1 across different cell lines. **(C)** IL4I1 expression comparison in normal versus neoplastic patient samples. **(D)** Effects of BCAA deprivation on tumor growth assessed via the CCK8 assay in 769-P cells. **(E)** Effects of BCAA deprivation on tumor growth assessed via the CCK8 assay in ACHN cells. **(F)** CCK8 assay assessing the cytotoxicity of sunitinib combined with BCAA deprivation in 769-P cells. **(G)** CCK8 assay assessing the cytotoxicity of sunitinib combined with BCAA deprivation in ACHN cells. **(H)** Plate cloning experiment demonstrating tumor growth following IL4I1 knockdown. **(I)** Effects of IL4I1 knockdown on tumor migration. **(J)** Physical subnetwork analysis of IL4I1 and branched-chain amino acid-related protein complexes. **(K)** Correlation of IL4I1 expression with other proteins in the CPTAC-KIRC dataset. **(L)** Correlation analysis of protein expression between IL4I1 and ACADB. **(M)** Correlation analysis of IL4I1 and AUH protein expression. **(N)** Impact of IL4I1 knockdown on the mRNA expression of branched-chain amino acid-related genes, including AUH. **(O)** Effects on BCKAs concentration 24 h post IL4I1 knockdown. **(P)** Effects on BCKAs concentration 72 h post IL4I1 knockdown.

We further investigated the relationship between IL4I1 and tumor progression. We analyzed the effects of high and low IL4I1 expression on various cell subsets (Figure 9A), finding that elevated IL4I1 expression significantly impacted tumor cells and macrophages. Verification of IL4I1 expression levels in cells and tissues revealed that IL4I1 is highly expressed in ccRCC (Figures 9B,C). Functional analyses suggested that IL4I1 primarily facilitates antigen processing and presentation, particularly concerning exogenous peptides and polysaccharide antigens presented via MHC class II (Supplementary Figure S1A). Additionally, high IL4I1 expression promotes multiple tumor progression phenotypes, including enhanced cell cycle activity, epithelial-mesenchymal transition (EMT), inflammation, invasion, and metastasis (Supplementary Figure S1B). Enrichment analysis indicated that IL4I1 inhibits the degradation of branched-chain amino acids, the tricarboxylic acid cycle, and other aspects of energy metabolism (Supplementary Figures S1C,D).

To further assess the role of BCAA in tumor growth, we conducted experiments using BCAA deprivation in conditioned media. CCK-8 assays demonstrated that BCAA deprivation significantly inhibits tumor growth (Figures 9D,E). Interestingly, BCAA deprivation also enhanced the tumor-killing effect of sunitinib to some extent (Figures 9F,G). Furthermore, plate cloning results indicated that IL4I1 knockdown inhibited tumor growth (Figure 9H). Transwell assays confirmed that IL4I1 knockdown significantly reduced tumor invasion and metastasis (Figure 9I).

We analyzed the correlation between IL4I1 and branched-chain amino acid-related genes (Figure 9J, Supplementary File), focusing on highly correlated proteins that were expressed at low levels in tumors (Figure 9K). Notably, AUH, ACADB, and IL4I1 exhibited negative correlations (Figures 9L,M), suggesting a possible regulatory relationship. We constructed stable knockdown strains and verified the regulatory relationship between IL4I1 and genes involved in branched-chain amino acid catabolism (Figure 9N). IL4I1 increased the mRNA expression levels of AUH but did not affect BCKDBHA and BCKDBHB, while reducing the expression levels of both BCAT1 and BCAT2. We further examined BCKA concentrations at different time points and found a slight decrease at 24 h, followed by an increase at 72 h (Figures 9O,P). This dynamic change likely reveals feedback regulation within the metabolic network triggered by IL4I1 knockdown. The early decrease (24 h) in BCKA levels may reflect the suppression of downstream signaling, potentially involving the AHR-BCAT axis, upon IL4I1 loss, leading to reduced BCKA generation. The subsequent accumulation (72 h) likely results from a compensatory feedback inhibition of the BCKDH complex—the key enzyme for BCKA oxidation—potentially mediated by the BCKDK kinase or other regulatory mechanisms, ultimately blocking BCKA consumption. Collectively, our functional experiments demonstrate a clear pro-tumorigenic role of IL4I1. This aligns with the clinical trend observed in our cohort, where high IL4I1 expression is associated with advanced tumor stage (Figure 3K), indicative of disease progression. While a complex statistical signal was observed in the Cox model (Figure 3C), the predominant biological function of IL4I1 supports its role as a driver of ccRCC progression. Additionally, we explored the mechanism underlying the high expression of IL4I1 in tumor tissues. Through pan-cancer e-QTL co-localization analysis, we found that IL4I1 and rs1290754 in renal cell carcinoma share a genetic causal variation (Supplementary Figures S1A,B). The predominant mutation type in IL4I1 was identified as a missense mutation, followed by nonsense mutations. The main type of single nucleotide variation was single nucleotide polymorphism (Supplementary Figure S2C). Notably, methylation changes in the CpG island region were statistically significant in KIRC, suggesting that the methylation status of this region may play an important role in the occurrence and progression of KIRC (Supplementary Figure S2D). ATAC-peak analysis of the Spearman correlation between IL4I1 and transcription factors (Supplementary Figure S2E) indicated that HIF1A could be a key regulatory transcription factor. Further correlation analyses using datasets GSE167573 and ICGC-EU (Supplementary Figures S2F,G) demonstrated a positive correlation between IL4I1 and HIF1A. Additionally, prior research indicated that knocking down SFMBT1 can lead to decreased IL4I1 expression (Supplementary Figure S2H).

## 4 Discussion

Clear cell renal cell carcinoma (ccRCC) is characterized by the signature “clear cell” phenotype that arises from abnormal lipid deposition in the cytoplasm. In contrast to fatty acid metabolism, the mechanisms underlying BCAA metabolism remain unclear. Although BCAA metabolism has been shown to influence the progression of tumors such as breast cancer and melanoma through pathways like mTOR (Zhang and Han, 2017; Sheen et al., 2011), this study highlights its unique clinical relevance in ccRCC. A comparison of treated and untreated patients revealed significant differences in BCAA metabolic characteristics related to tumor progression and treatment response (Figures 2A,J). Specifically, most BCAA metabolism-related genes are downregulated during tumor progression, while BCAT1 expression significantly increases with disease stage. This gene has been identified as a key cancer promoter and prognostic marker in various cancers, including breast cancer and leukemia (Hattori et al., 2017). While BCAT1 upregulation is a common feature in several cancers, the widespread downregulation of BCAA catabolic genes we observed in ccRCC more closely resembles the metabolic landscape of hepatocellular carcinoma than that of pancreatic or lung cancers, highlighting the tissue-specific nature of BCAA metabolic rewiring (Brown et al., 2026). Most notably, ACSF3 expression was significantly upregulated in the PD-1 immunotherapy-responsive group. ACSF3 regulates metabolic flux and mitochondrial protein malonylation by catalyzing the conversion of malonic acid to malonyl-CoA, which may profoundly impact tumor energy metabolism and the immune microenvironment (Lombard and Zhao, 2017). These findings underscore the central role of BCAA metabolic reprogramming in ccRCC progression and provide new targets for developing therapeutic prediction systems based on BCAA metabolic markers.

We employed 108 combinations of 10 machine learning algorithms for variable selection and model development to assess Amino acid Individualized Signature (AS). The TCGA dataset served as the training set, while the remaining datasets were utilized as the validation set. The results indicated that the random survival forest (RSF) algorithm exhibited the best performance (Figure 3D). To investigate the impact of different genes on model predictions, SHAP analysis was conducted on the RSF model, revealing that the top five significant genes were ACADM, PCCA, ACADSB, IL4I1, and ALDH3A2—each of which has been linked to poor prognoses in prior studies (Lin et al., 2023; Lv et al., 2024; Liu et al., 2021; Zhou et al., 2024). Furthermore, we examined the expression trends of these genes across various stages of TCGA-KIRC, finding that IL4I1 expression levels progressively increased with advancing stage, suggesting its crucial role in the disease progression of ccRCC (Figure 3K).

Based on AS scores, we classified ccRCC patients into subgroups, discovering that those with high AS scores had worse prognoses. In the high-score group, AS scores positively correlated with clinical progression and were identified as independent risk factors. In first- and second-line treatments, anti-angiogenic therapies did not exhibit significant differences between high-score and low-score groups; however, mTOR inhibitors in second-line treatment demonstrated greater predicted drug sensitivity in the high-score subgroup (Figures 4I–L). Regarding tumor biology, patients in the high-score group showed reduced activity in several metabolic processes, including the degradation of branched-chain amino acids and fatty acids (Figure 5D). In our phenotypic analysis of tumor progression, we noted that the inhibition of angiogenesis in high-score patients may contribute to their insensitivity to anti-angiogenic drugs (Figure 5E). Immune infiltration analysis indicated that B cells, CD4T cells, and CD8 T cells were predominantly present in high-score patients, with macrophages being the main myeloid cells (Figure 5F). This comprehensive analysis, which integrates clinical, transcriptomic, metabolic profiles, and immune cell infiltration, provides a valuable framework for personalized treatment in these two patient subgroups.

Recognizing the inherent heterogeneity among patients, we constructed Amino acid Individualized Signature (AIS) based on differentially expressed genes from AS score subgroups, as these may not fully capture individual conditions. Our results demonstrated that high AIS scores are associated with poor prognosis, while low AIS scores correlate with earlier clinicopathological stages (Figure 6C). In immunotherapy, low AIS score patients tended to exhibited relatively high expression of CD274(PD-L1), whereas those with high AIS scores commonly expressed elevated levels of PDCD1, CTLA4, and LAG3 (Figures 6J–M). We further explored the potential relevance of the AIS score in an external melanoma immunotherapy cohort, where analysis suggested that high AIS score patients may poorly responded to PD-1 inhibitor treatment, reflecting current immunotherapy realities where some patients initially respond well but later experience off-target effects, highlighting the need for better combinatorial treatment strategies (Barata and Rini, 2017). These findings, derived from a non-ccRCC cohort, are exploratory and require validation in ccRCC. In the context of targeted therapy, our analysis suggested that high AIS score patients might be more prone to inhibit Cuproptosis (Cobine and Brady, 2022). Notably, pRRophetic algorithm indicated that Cuproptosis inducer, Elesclomol, might have higher predicted sensitivity in patients with high AIS scores (Figure 7D), while the predicted sensitivity to common targeted drugs like axitinib showed no significant differences, thus offering potential clues and research directions for treating patients who have failed conventional first-line therapies (Figures 7E–G).

This study found that IL4I1 is closely related to ccRCC disease progression and prognosis, and its high expression is closely linked to immunotherapy response. Previous studies have shown that IL4I1 is associated with lipid metabolic reprogramming in ccRCC (Li et al., 2023). In addition, as a metabolic immune checkpoint, IL4I1 can activate aryl hydrocarbon receptor (AHR), bypass immune checkpoint blockade (ICB), and play an important role in immunosuppression and tumor microenvironment shaping in other tumors (Sadik et al., 2020). By analyzing the ccRCC spatial transcriptome data, we found that IL4I1 was highly expressed in both tumor cells and myeloid cells (Figure 8A). Interestingly, in untreated patients, single-cell data suggest that high IL4I1 expression increases tumor cell fraction while decreasing myeloid cell fraction (Figure 9A). Notably, advanced ccRCC is characterized by the concurrent presence of terminally exhausted CD8 T cells and M2-like macrophages that express specific ligands and receptors to support T cell dysfunction and M2-like polarization (Braun et al., 2021). These observations highlight the regulatory role of IL4I1 in the development and progression of ccRCC, and suggest that the prognostic value of IL4I1 in the interaction between amino acid metabolism and immune regulation in ccRCC warrants further investigation.

We further investigated the effects of IL4I1 and BCAAs on ccRCC tumor growth. Our findings indicate that BCAA blockade inhibits tumor growth and enhances sensitivity to the antiangiogenic drug sunitinib (Figures 9D–G), suggesting potential therapeutic applications. Similar studies have also been conducted in pancreatic cancer (Wang et al., 2024).This provides a promising new direction for treatments such as targeted metabolic reprogramming. Additionally, we examined the impact of IL4I1 knockdown on the expression of BCAA-related genes. Knockdown of IL4I1 led to an increase in AUH mRNA expression, while having no significant effect on BCKDBHA and BCKDBHB; however, the expression level of BCAT1 decreased (Figure 9N). AUH encodes methylglutaconyl-CoA hydratase, an enzyme in the leucine degradation pathway whose activity can be regulated to impact BCAA catabolic flux (Wang et al., 2025; Sivanand and Vander Heiden, 2020). It is noteworthy that IL4I1, an L-amino acid oxidase, directly targets aromatic amino acids (e.g., tryptophan) rather than BCAAs. Based on established literature, we hypothesize that IL4I1 may promote BCAA catabolism indirectly by activating the AHR signaling pathway via metabolites like kynurenine, which could lead to the upregulation of BCAT1/2, thereby forming a putative ‘IL4I1-AHR-BCAT’ regulatory axis (Sadik et al., 2020; Zeitler and Murray, 2023; Campesato et al., 2020). This proposed mechanism offers a plausible explanation for how IL4I1 influences BCAA metabolism. The observed accumulation of BCKA at 72 h (Figure 9P) further suggests that sustained IL4I1 loss triggers a secondary feedback inhibition downstream in the catabolic pathway.

The mechanism underlying the abnormally high expression of IL4I1 was investigated. At the genomic level, pan-cancer e-QTL Bayesian co-mapping analysis revealed a shared genetic causal variation between IL4I1 and rs1290754 in renal cell carcinoma (RCC), identifying the single nucleotide variation as a single nucleotide polymorphism (SNP) (Supplementary Figure S2A). In the epigenetic analysis, we integrated ATAC data to examine the transcription factors associated with IL4I1, with correlation analysis indicating that HIF1A serves as the key regulatory transcription factor (Du et al., 2017; Hoefflin et al., 2020) (Supplementary Figures S2E–G). Notably, the mutational inactivation of the VHL gene represents the earliest genetic event in most ccRCC, leading to an accumulation of HIF-1α and HIF-2α transcription factors. HIF-1α not only regulates glycolysis but is also closely linked to fatty acid metabolism, potentially exerting a significant impact on amino acid metabolism. In addition to HIF1α, SFMBT1 functions as another important transcriptional repressor in VHL-depleted ccRCC (Liu et al., 2020). Furthermore, results from transcription factor knockdown experiments revealed that silencing SFMBT1 led to a decrease in the expression level of IL4I1 (Supplementary Figure S2H).

Our study has several limitations. First, most of the data were sourced from public databases. Although the model was effectively developed and validated in both internal and external training sets, additional validation of its stability in our center or other multicenter cohorts would be beneficial. Second, we were unable to analyze key clinical factors (e.g., surgery, chemoradiotherapy, and radiation therapy) that may influence immune responses and drug sensitivity outcomes. Third, drug sensitivity was inferred computationally (pRRophetic) and requires biological or clinical validation. Third, key findings rely on computational inference and require further validation. The predicted drug sensitivities were derived from transcriptomic data (pRRophetic) and need biological or clinical confirmation. Similarly, the transcriptome-based inference of BCAA metabolic activity has inherent constraints. The observed discrepancy between pathway activity scores and the expression of individual genes underscores the complexity of metabolic regulation and highlights the need for validation using metabolomics or stable isotope tracing in future studies. Finally, while investigating the experimental mechanisms of IL4I1, it will be essential to conduct further *in vivo* and *in vitro* experiments to comprehensively elucidate the integrated role of branched-chain amino acid metabolism in ccRCC.

## 5 Conclusion

In conclusion, our integrative analysis reveals that BCAA metabolic reprogramming is a hallmark of ccRCC. We developed the AS and AIS prognostic signatures to decode this heterogeneity and pinpointed IL4I1 as a key driver of BCAA catabolism and tumor growth, offering a novel target for therapeutic intervention.

## Data Availability

The original contributions presented in the study are included in the article/Supplementary Material, further inquiries can be directed to the corresponding authors.
