# Supplementary material for: Machine learning-derived AS and AIS scores leverage BCAA metabolism and IL4I1 activity for prognosis and tailored therapy in ccRCC
Source: Front Cell Dev Biol. 2026 Feb 25;14:1720910. doi: 10.3389/fcell.2026.1720910 (PMC12978018; doi:10.3389/fcell.2026.1720910)
Supplement: Supplementary file 2 [file DataSheet1.docx]

Supplementary Material

**Supplementary Materials:** supplementary material: List of higher IL4I1 associated proteins and article associated Primer sequences.

## Supplementary Figure 1. Functional analysis of IL4I1


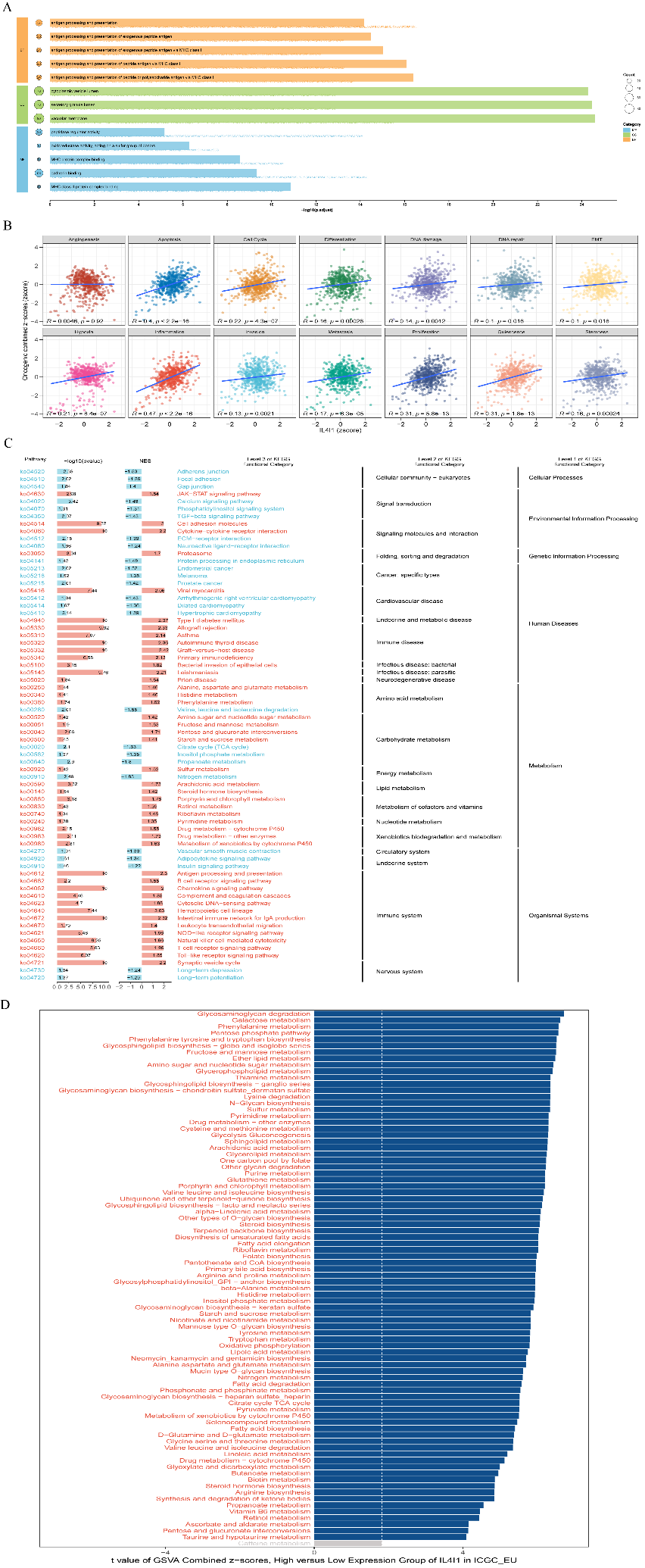


**Supplementary Figure2：Upstream gene analysis of IL4I1**


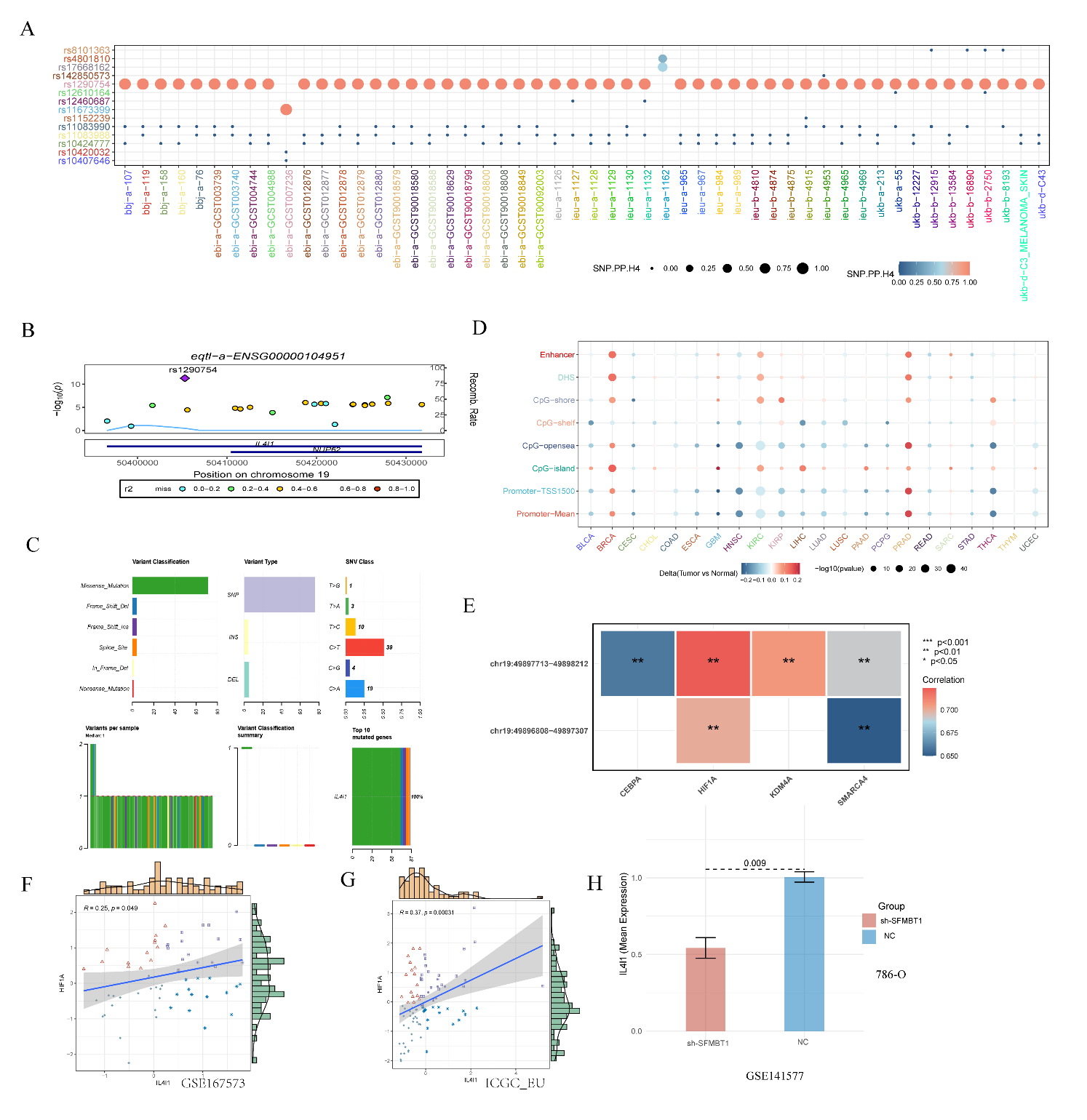


**Supplementary Figure3：Schematic of the lentiviral shRNA expression vector and sequences used for IL4I1 knockdown.**

**Schematic diagram of carrier**


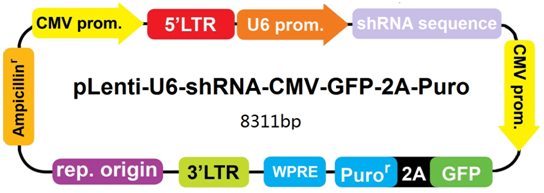


**IL4I1_shRNA:**

Target a：GCCAAGACCCCTTCGAGAAAT TTCAAGAGA ATTTCTCGAAGGGGTCTTGGC

Target b：GGCTGCAGAAAGGCGATGAAG TTCAAGAGA CTTCATCGCCTTTCTGCAGCC

Target c：CCAAGGTGTTCCTAAGCTTCC TTCAAGAGA GGAAGCTTAGGAACACCTTGG

**Control:**

**Scrambled shRNA:** GGGTGAACTCACGTCAGAATTCAAGAGATTCTGACGTGAGTTCACCC
